# Transient Presyncope Secondary to Posterior Descending Artery Occlusion

**DOI:** 10.1155/2011/792938

**Published:** 2011-08-18

**Authors:** Andrew D. Moffat, Jamal T. Al-Khatib, Jennifer Michael, Vien X. Nguyen

**Affiliations:** Graduate Medical Education Faculty, Kingman Regional Medical Center, 3269 Stockton Hill Road, Kingman, AZ 86409, USA

## Abstract

We describe the case of a 64-year-old male initially presenting with presyncope and bradycardia, without any anginal symptoms or objective evidence of myocardial ischemia. A stress test induced no physical symptoms but revealed a left bundle branch block with multiple preventricular contractions on electrocardiogram. Subsequent catheterization revealed severe obstructive disease throughout the coronary arteries. He was treated percutaneously on two separate heart catheterizations. The presyncope and bradycardia resolved after reperfusion of the posterior descending artery.

## 1. Introduction

Transient symptomatic bradycardia secondary to posterior descending artery (PDA) occlusion is rare. Lin and Cheng [1] reported a patient with acute-onset dizziness due to total occlusion of left circumflex (LCx) artery without objective evidence of myocardial infarction at initial presentation. We herein present a similar case of transient presyncope due to a nearly occlusive PDA in a patient without any evidence of cardiac ischemia. Early and accurate diagnosis of this serious condition is crucial for prompt and successful coronary revascularization.

## 2. Case Presentation

A 64-year-old white male with a history of uncontrolled hypertension, hyperlipidemia, and posttraumatic stress disorder presented to our medical center with five presyncopal episodes in the previous 24 hours. The patient had experienced similar events a few times a month over the past 3 years, but lately they had become more frequent and intense. He described these events as dizziness and lightheadedness with balance difficulty lasting only a few seconds, forcing him to sit down for 1 to 2 minutes at a time. The patient denied chest pain, palpitations, shortness of breath, or loss of consciousness. 

The vital signs revealed a blood pressure of 158/72 mmHg, pulse of 51, and a respiratory rate of 16. The serial cardiac enzymes were unremarkable. On telemetry, there were several episodes of asymptomatic sinus bradycardia, dropping as low as 41 beats per minute. Computed tomography of the head, magnetic resonance imaging of the brain, carotid ultrasound, and two-dimensional echocardiogram were essentially normal. Cardiology consultation was obtained for sinus bradycardia, and the patient underwent a technetium (99mTc) sestamibi treadmill stress test.

**Figure 1:**
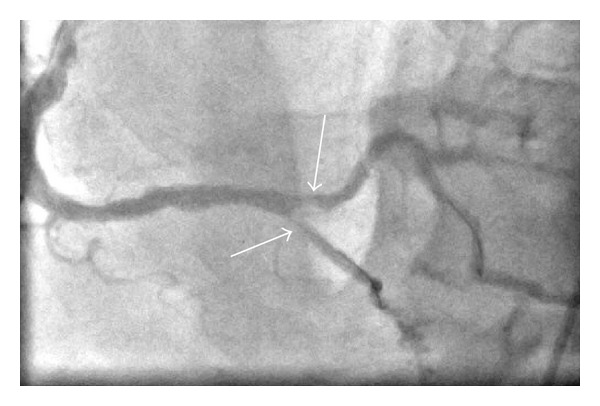
Angiogram showing 90% occlusion of PDA (left arrow) and 70% of distal RCA (right arrow).

During the stress test, the electrocardiogram showed a left bundle branch block (LBBB) with multiple preventricular contractions (PVCs). The patient had no chest pain or presyncope throughout the test. A subsequent cardiac catheterization identified multiple stenoses (70–90%) in the left anterior descending artery (LAD), total occlusion of the nondominant LCx, 70% stenosis in the distal right coronary artery (RCA), and 90% stenosis in the PDA (see Figure 1).

As the patient's disease was mostly at the apex, the optimal treatment was balloon angioplasty with stent placement. Five drug-eluding stents were successfully placed in the LAD. The patient's heart rate was still near 40 beats per minute, and the patient was transferred to the intensive care unit for close monitoring. After two days without improvement, he underwent a second catheterization where one stent was placed in the distal RCA and another in the PDA. Following the second procedure, his bradycardia resolved, maintaining a heart rate in the 70's. After five months of followup, he continues to be asymptomatic.

## 3. Discussion

We describe a unique presentation of severe coronary artery disease (CAD) without angina or initial objective evidence of infarction. A similar case has previously been reported, where the symptoms resolved after LCx angioplasty [1]. Our patient's symptoms resolved after stent placement in the PDA. Approximately 20% of patients with symptomatic bradyarrhythmias have coexistent CAD [2]. As the sinus and atrioventricular nodes receive blood supply primarily from the RCA and secondarily from LCx, compromise of flow from either of these vessels to these nodes can cause sinus bradyarrhythmias [3]. We assumed that the cause of transient presyncope in this patient was due to unstable hemodynamics caused by such bradyarrhythmias. During the stress test, our patient was completely asymptomatic; only PVCs and a LBBB were observed. This could be explained by coronary collateral vessels, as there is a correlation between bradycardia and collateral vessel development in patients with obstructive CAD. However, we were unable to confirm this due to angiography's limitation in detecting collaterals less than 50 micrometers in diameter [4]. CAD should be on the differential diagnosis list in the initial workup of patients with transient presyncope and “asymptomatic” bradycardia because missing this diagnosis may have detrimental consequences.

## References

[1] Lin CF, Cheng SM (2006). Symptomatic bradycardia due to total occlusion of left circumflex artery without electrocardiographic evidence of myocardial infarction at initial presentation. *Texas Heart Institute Journal*.

[2] Hsueh CW, Lee WL, Chen YT, Ting CT (2001). The incidence of coronary artery disease in patients with symptomatic bradyarrhythmias. *Japanese Heart Journal*.

[3] Serrano CV, Bortolotto LA, Cesar LA (1999). Sinus bradycardia as a predictor of right coronary artery occlusion in patients with inferior myocardial infarction. *International Journal of Cardiology*.

[4] Patel SR, Breall JA, Diver DJ, Levy AP (2000). Bradycardia is associated with development of coronary collateral vessels in humans. *Coronary Artery Disease*.

